# Unveiling the dual potential of microalgae and seaweed biomass for sustainable biofuel production: a review

**DOI:** 10.1039/d5ra04845a

**Published:** 2025-09-18

**Authors:** D P Krishna Samal, Lala Behari Sukla

**Affiliations:** a Biofuels and Bioprocessing Research Center, Institute of Technical Education and Research, Siksha ‘O’ Anusandhan (Deemed to be University) Bhubaneswar 751030 India lalabeharisukla@soa.ac.in

## Abstract

Fossil fuels account for 82% of the overall energy supply, meeting rising global energy demand. Oil accounts for 35%, coal for 29%, and natural gas for 24%. However, fossil fuels are limited and largely contribute to environmental damage. Global energy consumption is expected to rise by 2040, emphasizing the urgent need for sustainable energy solutions. Although renewable energy technologies can meet this need, they account for less than 13% of the overall energy supply. Algae have received attention as a prospective source of third-generation biofuels such as biodiesel, bioethanol, biogas, and biohydrogen. They grow quickly, consume less area, absorb more CO_2_, and do not compete with food crops. Algae-based biofuels are carbon-neutral, balancing CO_2_ emissions throughout production and usage. Algae can also be produced in non-arable regions, saving agricultural land for food production. This review focuses on the biofuel production potential of both microalgae and macroalgae. It examines the benefits, limits, and research gaps. Strategies for increasing algae-based biofuel generation are highlighted. Addressing these issues can harness algae's potential as a renewable energy source, contributing to sustainable energy solutions and lowering environmental concerns.

## Abstract 2

Microalgae and seaweed biomass for sustainable biofuel production.

## Graphical abstract

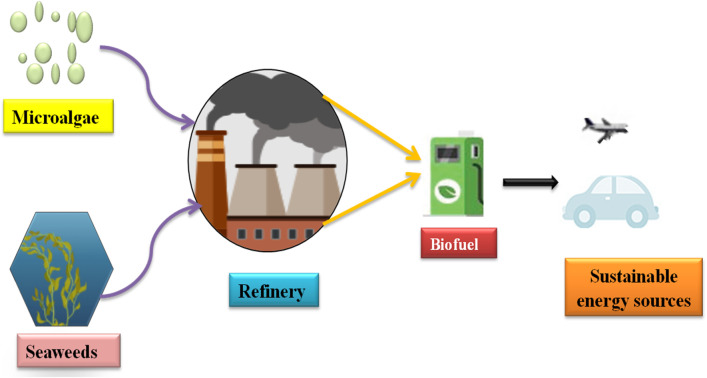

## Introduction

Global energy demand is rapidly increasing and fueled by economic and population growth.^1^ Fossil fuels such as oil, coal, and natural gas currently dominate the energy supply. Most of the world's energy currently comes from fossil fuels, which are limited resources that still play a significant role in meeting our energy needs.^2^ Fossil fuels account for 82% of the world's total energy consumption.^3^ Oil constitutes the largest share at 35%, coal at 29% and natural gas at 24%.^4,5^ Globally, the industrial sector is the largest consumer of energy. The industrial sector consumes approximately 37% of the energy. 28% of energy is used in the transportation sector, 22% in household consumption, and 13% in commercial sectors. However, this heavy dependence on fossil fuels raises serious concerns about long-term sustainability. However, their finite nature and environmental impact necessitate a shift towards sustainable alternatives. The transition from fossil fuels to renewable energy sources is a multifaceted process. Fossil fuels currently dominate because of their established supply chains and existing infrastructure. Shifting to renewables such as biofuel and bioenergy requires significant technological innovation. In 2040, global energy consumption is expected to increase significantly, being 15% lower in the 450 scenario and 10% higher in the current policy scenario.^6^ This underscores the urgency of addressing future energy needs sustainably. Hence, the world is witnessing a significant shift towards renewable energy sources. Although renewable energy has the potential to meet global energy demands with existing technologies, it currently contributes less than 13% of the total energy supply.^7^ Despite this, advancements in renewable energy technologies are opening new avenues for sustainable energy production.

Microalgae have emerged as a viable feedstock for biofuels such as biodiesel, bioethanol, biogas, and biohydrogen.^8^ Third-generation biofuels, made from microalgae and marine macroalgae (seaweed), are among the most promising renewable energy sources. Microalgae offer several advantages, including faster growth rates, lower land usage, and higher CO_2_ absorption and uptake rates.^9^ Algae-based biofuel can be cultivated in areas unsuitable for conventional agriculture. This preserves arable land for food crop production.^10^ No fertilizers are used and no competition for food resources exists for algal cultivation. These biofuels have many advantages over 1st and 2nd generation biofuels. The carbon neutrality of algal biofuels is a significant advantage. When algal biomass grows at the same rate as consumption, it reduces carbon emissions from fuel burning.^11^ This balanced growth cycle and consumption does not increase overall CO_2_ in the atmosphere. Despite the growing emphasis on renewable energy, the current contribution from these sources is limited to the total energy supply. So, algal biofuels meet the renewable energy requirement and reduce their environmental impact. So, microalgae and macroalgae are recognized as viable energy sources for eco-friendly biofuel production.^12^

This review examines the potential of using microalgae and macroalgae to manufacture biofuels. The primary focus is on their ability to produce biofuels, current restrictions, and areas that require additional exploration. The study underlines the importance of investigating successful tactics. The study underlines the necessity of looking into practical strategies to increase the production of fuel, biogas, and bioethanol from microalgae and macroalgae. Solving these research and development obstacles may expose algae's potential as a renewable energy resource.

## An overview of biofuels: types and applications in sustainable energy

For quite a while, biofuels have been utilized as a renewable energy resource. They are mainly derived from agricultural products such as plants, wood, seeds, and biomass. Biofuels exist in three states: liquid, solid, and gas.^13^ These biofuels offer a wide range of power generation and transportation applications. The main products of the liquid biofuels are biodiesel and bioethanol. These are derived from natural biomass and are energy-dense.^14^ Solid biofuels can be produced from agricultural products; examples include animal dung, charcoal, and fuelwood, which are non-fossil feedstocks.^15^ These solids are mainly used for the generation of heat and energy. They are considered suitable substitutes for petroleum fuels because of their availability and friendly effects on the environment. More advanced techniques like pyrolysis and gasification are employed in manufacturing gaseous biofuels like biogas and biohydrogen.^16^ The use of biofuels for combustion has no net negative impact on carbon dioxide in the atmosphere. Hence, plants can take in carbon dioxide while performing photosynthesis, which sort of evens the burning emission.^17^ Some of the common biofuels are:

### Bioethanol

Bioethanol production began in the 1800 s. It is extensively utilized as a partial replacement for gasoline because of its fuel efficiency benefits.^18^ It is also a safer substitute for methyl tertiary butyl ether (MTBE) as a fuel additive. Bioethanol is a sustainable energy source made by fermenting sugar. It is primarily produced by fermenting sugars from crops like sugarcane, sweet sorghum, and sugar beet. The United States and Brazil produce over half of the global ethanol supply, primarily from crops like maize and sugarcane.^19^ However, bioethanol production from corn faces challenges like competition with food crops for agricultural land. Bioethanol from straw, wood chips, and forestry waste offers a sustainable alternative. However, its production involves complex pretreatment and hydrolysis processes.^20^ Advanced technologies, such as consolidated bioprocessing (CBP), aim to simplify production and reduce costs. Ethanol's applications extend beyond fuel, including raw materials for chemical processes and hydrogen production. Apart from all these, bioethanol can be produced from algae through fermentation. Algal metabolic activity is affected by pH and temperature, which alters enzyme activity and microbial fermentation processes.^21^ The fermentation, is usually carried out at temperatures between 25–35 °C and under a pH range of 4.5–5.5 by algae. Microalgae usually require a temperature of 30–35 °C, but macroalgae require 25–30 °C for bioethanol production.

### Biodiesel

Biodiesel is produced by transesterifying fatty acid methyl esters sourced from vegetable oils, animal fats and algae.^22^ While commercially established, biodiesel has competitors such as hydrocracked biodiesel, which is less developed but holds future potential. Feedstocks for biodiesel production include oil crops, recycled oil, animal fats, and algae.^23^ The quality and cost of biodiesel vary depending on the pretreatment and treatment processes applied to these feedstocks. Biodiesel production from algae faces several challenges, particularly during lipid extraction and transesterification. The fatty acid composition of the algal lipids significantly impacts the quality of the resulting biodiesel. Converting lipids into biodiesel depends on several factors, including the catalyst type, reaction time, and methanol-to-oil ratio.

### Bio-oil

Bio-oil is a dark brown liquid produced by heating oxygen-free biomass algae. It has trace levels of solids and chemical substances in water. Microalgae are a promising feedstock for bio-oil due to their fast growth, high lipid content, and ability to grow in freshwater saltwater and wastewater.^24^*Chlorella vulgaris*, *Nannochloropsis* and *Dunaliella tertiolecta* are ideal due to their high oil yields.^25^ Thermochemical methods like pyrolysis are used to convert microalgal biomass into bio-oil. It can be burned to produce heat and electricity. After refinement, it can be transformed into fuels like green gasoline or biodiesel.

### Biohydrogen

Biohydrogen is an eco-friendly energy source produced by microalgae using light and water. Microalgae can produce biohydrogen through various methods, such as dark fermentation, photofermentation, and biophotolysis.^26^ In biophotolysis, light energy is used to split water molecules into hydrogen and oxygen through the enzyme hydrogenase. Microalgae like *Chlamydomonas reinhardtii* can directly produce hydrogen. In dark fermentation, biohydrogen is produced by algae breaking down organic molecules without light.

### Biogas

Biogas has been produced from biomass for centuries. The use of biogas for electricity has expanded rapidly in recent decades. Biogas is produced through anaerobic digestion. Microorganisms degrade organic waste in the absence of oxygen during anaerobic digestion.

This process mimics the natural formation of underground natural gas over millions of years. The process involves multiple stages: hydrolysis of organic molecules into smaller units, acidogenesis to volatile fatty acids, acetogenesis, and finally, methanogenesis.^15^ It produces a gas mixture of methane (55–90%) and carbon dioxide, with minor impurities like hydrogen sulfide and mercaptans. Biogas is a sustainable energy source with substantial heating capacity when methane exceeds 50%. Algal biomass can improve total bioenergy recovery while reducing waste by integrating with the current anaerobic digestion. It can balance the carbon-to-nitrogen ratio, thereby improving biogas yield. However, the significant challenges are the high water content and the recalcitrant nature of algal cell walls. Effective mechanical, thermal, or enzymatic pretreatment methods are crucial to improve biomass breakdown and enhance methane production. Biogas production is widely adopted in agriculture-intensive countries like India, China, and Brazil.^27^ It is also applied in food processing, pulp and paper, and household waste treatment. It reduces environmental pollution by lowering NOx and particulate emissions during combustion.^28^

## Evolution of biofuels: advances across four generations

Biofuels are classified into four generations based on their feedstock origin. First-generation (G1) biofuels are derived from food crops and edible oils^29^ other aquatic biomas (Table 1). These fuels include biodiesel, bio-alcohols, and vegetable oils, which are produced through methods like transesterification, anaerobic decomposition, and pyrolysis.^15,30^ First-generation biofuels are widely used globally. Countries like the U.S., India, and Brazil rely on crops like corn and sugarcane for ethanol production.^31^ However, concerns about G1 biofuels include their impact on food security and potential social conflicts due to land competition. This has led to debates about the sustainability of relying on edible crops for energy. In general, G1 biofuels need much water for crop cultivation. Hence, it contributes to soil deterioration and eutrophication by using much water for irrigation and processing, as well as chemical pesticides and fertilizers. Changes in land use, such as deforestation, can release large volumes of carbon dioxide, which can partially negate the greenhouse gas that G1 biofuels offer (Table 2). Greenhouse gases also increase due to the high energy requirements for growing, harvesting, processing, and transporting these crops.

**Table 1:** Different generations of biofuels and their sources

Generation	Source type	Examples of sources	References
1st generation (G1)	Food crops & edible oils	Corn, sugarcane, rapeseed, sunflower, soybean, palm, mustard, coconut, wheat germ	41–43
	Animal fats	Beef tallow, lard	44 and 45
	Nut oils	Almond, walnut, pistachio	46 and 47
2nd generation (G2)	Non-food biomass & inedible oils	Dry wood, corn stalks, wheat stalks, rice husks agricultural residues, sugarcane bagasse	48 and 49
	Waste oils	Used cooking oil, restaurant waste oil	50 and 51
	Inedible oil crops	Jatropha, neem, jojoba, mahua, rubber seed, babassu tree	52 and 53
	Animal wastes	Manure, animal processing waste	54 and 15
3rd generation (G3)	Microalgae	*Chlorella*, *Spirulina*, *Chlamydomonas*	55–58
	Macroalgae	*Saccharina*, sargassum *Laminaria*, *Gracilaria*, and *Ulva*	59 and 60
	Other biomass	Water hyacinth, duckweed, insects	23,61 and 62
4th generation (G4)	Genetically modified microalgae	*Phaeodactylum tricornutum* (increased TAG accumulation through *GPAT2* gene overexpression), *Neochloris oleoabundans* (co-expression of *NeoLPAAT1* and *NeoDGAT2* increased lipid content)	63 and 64

**Table 2:** Advantages, disadvantages and scalability of different biofuel generations

Biofuel generation	Disadvantages	Advantages	Scalability	References
G1	• Competes with food production	• Easily available crops	Scalability is higher, but it has limitations due to competition with foods	41, 42 and 46
	• Increase food price			
	• Contribute to deforestation as agricultural lands are limited			
G2	• Requires advanced technologies	• Made from non-food biomass	High scalability due to large feedstock availability	50–52
	• Energy-intensive	• Reduces food competition		
	• Costly	• Lower greenhouse gas emissions		
G3	• High cost	• Derived from algae, which has high energy yields	High scalability but high production costs	55, 59 and 60
	• Controlled environmental conditions needed for growth and harvesting	• Can be grown on non-arable land		
		• Require CO_2_ during production		
		• Lower greenhouse gas emissions		
G4	• Ethical and regulatory concerns	• Uses engineered organisms or synthetic biology to produce biofuels	Potential for high scalability but requires extensive research and regulatory approval	63 and 64
	• Not yet commercially viable	• Potential for higher yields and lower costs		

Second-generation (G2) biofuels are produced from non-food biomass sources, such as wood, plants, and agricultural waste.^32^ G2 biofuels can be produced from fermentation gasification and hydrothermal liquefaction (HTL).^33^ These techniques offer eco-friendly, long-term solutions. G2 biofuels have captured global attention because their potential for sustainable production enables them to steer clear of the potential deforestation and food security problems associated with fuels produced from food crops. Moreover, the production of animal fats and waste cooking oils supports the sustainability of renewable energy markets worldwide. G2 biofuels, such as syngas, ethanol, butanol, and biohydrogen. While G1 biofuels are prevalent today, they suffer from the challenge introduced by the need to utilize food crops to determine whether they can be sustainable in the long term. For these reasons, G2 biofuels use non-food biomass sources, minimizing the impact on food production while accounting for environmental concerns. According to Soares *et al.*, G2 biofuels utilize agricultural wastes and inedible oils, reducing waste and promoting a circular economy.^34^ The key concern of the G2 biofuels production processes is energy balance (Table 2). Production processes like pretreatment, harvesting, and enzymatic hydrolysis require substantial energy input. The net energy ratio indicates how much energy the operation returns by comparing the energy intake and output. A biofuel with a high net energy ratio generates more energy than it consumes.

Third-generation (G3) biofuels are primarily sourced from microalgae, macroalgae (known as seaweed), or other aquatic biomass^35^(Table 1). Due to their high lipid and carbohydrate content, these algae can be used as potential sources to produce biofuel. Using algae to produce methane was first proposed in the 1950s. The idea became very popular in the 1970s. This idea became well-known during the energy crisis. Since then, algae biomass has become an important source of third-generation biofuels. Algae species like *Chlorella* and *Spirulina* produce bioethanol and biodiesel.^36^ This biomass can be transformed into fuels and by-products through several procedures. Biochemical, thermochemical, and chemical methods are among these processes. This provides effective energy solutions without vying for agricultural land with food crops. Since algae absorb CO_2_ during their growth, algal biofuels also help to reduce greenhouse gas emissions (Table 2). A sustainable substitute for G1 and G2 biofuels is G3 biofuels.^33^ The market for G3 biofuels is expected to grow significantly as long as research and development continues. Because of their high polysaccharide content, marine algae species of seaweeds are very efficient at producing biogas.^37^ Compared to conventional crops, biogas from algae can produce significantly more fuel per hectare. In addition to bioethanol and biodiesel, algae-based biofuels include syngas, biohydrogen, and bio-oil. Genetically modified (GM) algae, photobiological solar fuels and electro-fuels are examples of advanced technologies used in fourth-generation biofuels (G4) (Table 1). GM algae are engineered to improve photosynthetic efficiency and increase light penetration. These technologies enable precise modification of targeted microalgal genomes to maximize biofuel yield. CRISPR/Cas9, TALEN, and ZFN helps in optimizing these modifications.^38,39^ Genetic modifications in microalgae also facilitate efficient oil extraction through processes like cell autolysis and product secretion systems.^39,40^ Biofuels harness solar raw materials, offering an abundant, cost-effective, and inexhaustible resource for long-term sustainability. Fourth-generation biofuels integrate advanced techniques to meet energy demands while minimizing environmental impact (Table 2). This innovative strategy marks a significant step in achieving sustainable and economically viable biofuel production. This innovative approach represents a significant leap toward sustainable and economically viable biofuel production.

Properly developing and deploying genetically altered algae strains requires engagement with regulatory organizations and adherence to international biosafety standards. Closed bioreactor systems and kill-switch genetic designs address the issue of genetically modified organisms (GMOs) being used in biofuel production (Table 2).

### Biofuel production pathways

Biofuel production pathways are generally categorized as biochemical and thermochemical processes.

### Biochemical conversion pathway

In the biochemical conversion pathway, living organisms such as bacteria and yeast convert algal biomass into biofuel.^65^ Anaerobic digestion, fermentation, and enzymatic transesterification are the major processes involved in biofuel production. This process often starts with enzymatic hydrolysis, where complex carbohydrates are broken down into simple sugars. Microbes ferment these sugars to produce bioethanol. Similarly, in the anaerobic process, organic matter in the absence of oxygen breaks down by microbes to produce biogas. With a relatively lower energy input, this pathway can produce biofuels efficiently, but it usually is slower than the thermochemical pathway.

### Thermochemical conversion pathway

In the thermochemical conversion pathway, heat is used to break down algal biomass. No microbes are included in this process.^66^ This method includes direct combustion, Pyrolysis and gasification. A wide variety of algal biomass can be used in this process. Its fast reaction speed and flexibility in producing different biofuels require high energy inputs.

### Factors affecting biofuel production and quality

#### Feedstock composition

The chemical compositions and physical nature of feedstock play a significant role in biofuel production yield and quality. Feedstocks that are high in carbohydrates are suitable for producing bioethanol and biogas. Biodiesel manufacturing requires high lipid content, such as triglyceride and fatty acid-containing feedstocks. Algae rich in oils are used to produce biodiesel through transesterification.^67^ Excess of fatty acids can form soap during transesterification, requiring intensive pretreatment. Another important factor is protein content, which is influenced particularly in biogas systems. Moderate protein levels can promote microbial growth in anaerobic digestion. Excessive protein may prevent ammonia production, lowering methane productivity.^68^

### Processing conditions

Processing parameters such as temperature, moisture content, retention time, and the type of enzymatic treatment play a vital role in determining the efficiency of biofuel production.^69^ The alcohol-to-oil ratio and the catalyst used in the synthesis of biodiesel have a significant impact on the quantity and quality of biofuel. High moisture levels need more energy for drying, decreasing the overall energy balance. However, anaerobic digestion is more effective with high moisture-content feedstocks. Temperatures over 800 °C during gasification operations encourage more hydrogen and syngas generation.^70^

### Algal biomass as a sustainable feedstock for biofuel production

Over the past decade, algae have garnered significant attention due to their economic potential in large-scale cultivation for biofuel production. Algae are majorly grouped into micro and macroalgae.^71^ They are versatile aquatic organisms capable of photosynthesis. Microalgae are single-celled and offer distinct benefits for biofuel production. Macroalgae are commonly known as seaweed.^72^ Approximately 700 species of marine algae are found in India, with around 60 species being commercially important for food, medicine, fertilizer, and processing phycolloids and chemicals. Algae's high biomass potential and ability to convert carbon dioxide make them a promising sustainable biofuel source.

### Microalgae

Being the third-generation biomass, microalgae is the one that is used mostly in conversion processes. Microalgae, with their diverse range of species, offer promising potential as biofuel sources.^73^ In India, microalgae are abundant along the coastal regions of Odisha, Goa, Gujarat, Tamil Nadu, and the Lakshadweep and Andaman and Nicobar Islands. India has a vast coastline and abundant microalgae species. Thus, producing third-generation biofuels from microalgae is a promising opportunity to meet the country's energy demands without competing with food crops or agricultural land. Microalgae-based biofuels show great potential as a sustainable energy source for developing nations. For developing countries, decentralized microalgae cultivation systems can offer localized energy production.

Lipid content in microalgae ranges from 20–50% of the dry weight and can reach as high as 80% under specific conditions. These organisms are highly efficient at using CO_2_ and play a significant role in global carbon fixation, primarily from marine microalgae. Algae grow rapidly, with some species doubling in at least six hours. The primary direct route to obtaining energy from macroalgae is through its anaerobic digestion (AD) to biogas of 60% methane.^74^ Anaerobic digestion is a natural biochemical process that converts organic matter into biogas without oxygen. This process has several advantages, including a high degree of organic matter reduction and the production of a solid remainder that can be used as an organic fertilizer for arable land. This method is well established and available for purchase in several nations, including the United States, France, the United Kingdom, and Ireland. Several species, such as *Botryococcus* spp., can naturally retain substantial amounts of oil, up to 50% of their dry weight being long-chain hydrocarbons. Through genetic development, scientists can use this variety to identify and improve biofuel strains.

### Microalgae cultivation mechanisms and its significant potential for biofuels production

Algae biofuel production has significant potential as a replacement for traditional fuels. Algae may grow swiftly in various settings and offer an environmentally benign, carbon-neutral solution. Diverse microalgae species have enormous potential for manufacturing environmentally friendly biofuels (Table 3). Creating biofuels from algae includes multiple important stages: growing algae, gathering or removing water, extracting oil, refining algal oil, and converting the oil into biofuels.^75,76^ Basic resources such as light, dissolved nutrients and CO_2_ are necessary for algal culture. Microalgae required an optimal temperature range of 20 to 30 °C, light intensity 100–200 μmol photons per m^2^ per s^1^, and carbon dioxide 1–5%. Microalgae cultivation for biofuels typically requires a substantial amount of water.^77^ Open pond systems necessitate significant water inputs. To minimize environmental impacts, strategies include recycling water, using non-potable water sources (*e.g.*, wastewater or saline water), and optimizing water management.

**Table 3:** Several species of algae are used to produce biofuels

Species	Lipid content	Types of lipid components	Mechanisms
*Chlorella vulgaris*	High	Triacylglycerol	High triacylglycerol (TAG) yield; nitrogen restriction boosts lipid production
*Tetraselmis striata*	High	Various fatty acid methyl esters (FAME)	Rapid growth and high lipid content; thrives in co-culture
*Auxenochlorella protothecoides*	Medium	Mixed lipids	Co-culture with *E. coli* increases biomass and lipids
*Scenedesmus quadricauda*	Medium	Fatty acid methyl esters	High biomass yield in glucose-enriched medium
*Nannochloropsis oceanica*	Medium-high	Fatty substances	Targeted for genetic engineering to enhance lipid yield
*Phaeodactylum tricornutum*	High	FAME, TAG	Overexpression of *GPAT2* gene results in TAG hyperaccumulation

Microalgae cultivation requires less land. It can be cultivated in brackish water bodies or on non-arable land. Algal cultivation can be classified into two: batch cultivation and continuous flow cultivation. Algae are introduced into containers with ample resources in batch culture, and their growth peaks in a sigmoidal pattern before running out of resources. Fresh medium is consistently added in continuous flow culture to keep a stable state, ensuring that the birth rate of algae matches the death rate. This method maintains a steady state for algal growth. Compared to batch systems, continuous flow systems offer more productivity, consistent biomass output, and control over growing conditions.^78^ However, constant mixing, aeration, temperature regulation, and nutrient supply are needed.

**Fig. 1:**
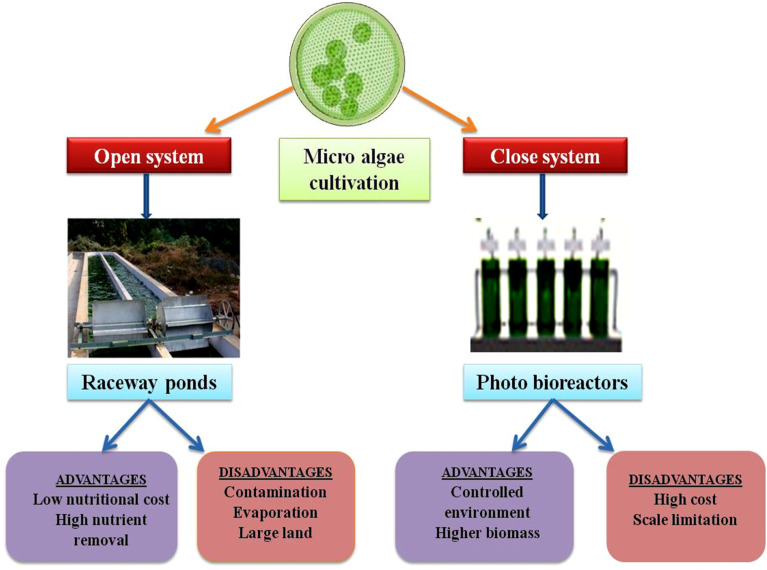
Microalgae cultivation system.

Regarding scalability, continuous systems are more suitable for industrial applications because they operate non-stop and produce consistent biomass yields.^78^ Scaling up requires careful system design to manage contamination risks and ensure uniform nutrient distribution. Despite higher operational costs, continuous flow cultivation is economically viable for large-scale applications. Three central culturing systems are in use: open pond systems, photobioreactors, and hybrid systems.^79^ Open pond systems are the most common but suffer from inefficiencies in mass and heat transfer (Fig. 1). In large-scale open pond systems, contamination in microalgae cultures poses a significant challenge to biofuel production. These contaminants can reduce algal growth, interfere with biofuel yield, and increase production costs.^80^ Controlled inoculation with high-performing algal strains and continuous monitoring of culture conditions is required to mitigate this risk. Water and nutrient media sterilization before microalgae inoculation is a necessary step. Regular cleaning and maintenance of the ponds and integrating semi-closed or closed-loop systems can limit contamination.^80^ Photobioreactors offer higher productivity rates but face challenges such as high costs and complexity (Fig. 1). Hybrid systems combine open ponds and photobioreactor systems. The hybrid system is most efficient for optimizing algal growth. In microalgal culture, advanced photobioreactors, including tubular and flat-panel reactors, offer benefits by improving light usage and lowering running costs.^81^ Flat-panel reactors have a large surface area. Thus, the exposure and penetration of light are higher, leading to increased algal growth. Because of its continuous flow, tubular reactors reduce the risk of photoinhibition.

**Fig. 2:**
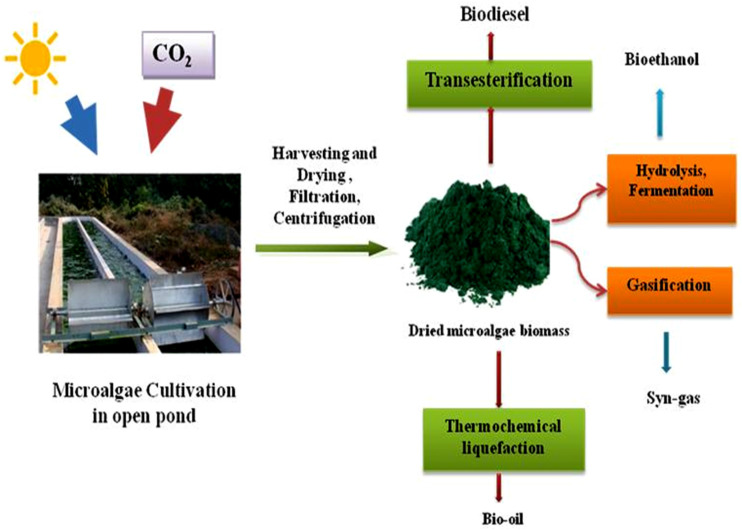
Mechanisms of extraction of biofuels from the microalgal biomass.

The cell walls of algae are composed of polysaccharides and cellulose.^82^ The cell wall must be disrupted to release the lipids. Methods like bead beating, microwave treatment, and ultrasound are commonly used.^83^ After algae is cultured, it must undergo a harvesting and dewatering process.^84^ This is crucial to access their lipid content, which is the primary source of biofuel. Harvesting typically involves filtration and centrifugation. Recently, some advanced techniques, such as flocculation and membrane filtration, have been explored for efficiency. Once dewatered, algae undergo lipid extraction to obtain oils that can be converted into biofuels.^85^ The production of algal oil is determined by the extraction method used. Solvent selection, polarity, temperature, and pressure significantly impact lipid yield and quality.^86^ The polarity of the solvent is crucial in the extraction of various lipids. Polar solvents like methanol or ethanol are better for phospholipids and glycolipids. Non-polar solvents like hexane extract neutral lipids. A combination of polar and non-polar solvents is employed for improved lipid recovery. High pressure enhances solvent penetration and lipid solubilization, leading to higher purity of lipids.^86^ Lipid solubility and solvent diffusivity are both strongly influenced by temperature. Higher temperatures improve extraction efficiency. Algal oil, produced by Soxhlet extraction or supercritical fluids, is a triglyceride that can be processed into biofuels.^87^ Solvent-free methods like supercritical fluid extraction (SCF) have been shown to extract high-quality oils without using environmentally hazardous chemicals. Supercritical fluid extraction requires high energy and costly equipment, making it less economically viable for large-scale operations. It also requires high pressure, typically 1000–5000 psi, to maintain the supercritical state of CO_2_. Traditional methods like solvent extraction and cold pressing are more cost-effective due to lower costs and result in lower lipid yield and quality. Overall, SCF is ideal for high-value products, but traditional methods remain cost-effective applications. Cyclopentyl Methyl Ether (CPME) and ethanol as supercritical fluids have been shown to extract more oil than the conventional CO_2_ method.^33^ This is a more environmentally friendly and effective way to get algae biofuel (Fig. 2).

### Transesterification

Transesterification in microalgae refers to the chemical reaction used to convert the lipids (typically triglycerides) extracted from microalgae into biodiesel.^88^ The reaction typically involves a triglyceride molecule reacting with an alcohol (commonly methanol) in the presence of a catalyst (acid, base, or enzyme). The general reaction equation for transesterification is as follows:Triglyceride + 3ROH → 3RCOOR′ + Glycerol

For high biodiesel yields, a molar ratio change between triglycerides and alcohol is essential. The production of biodiesel is often increased by a larger alcohol-to-oil ratio, such as 12 : 1. In a higher molar ratio, alcohol consumption also increases, leading to higher costs for the alcohol and additional processing steps to recover excess alcohol.^89^ Additionally, this may have a greater environmental impact, particularly if methanol is used. A lower molar ratio, like 6 : 1, may produce a lower biodiesel yield. It has slower reaction kinetics, as insufficient alcohol can lead to unreacted triglycerides. A lower molar ratio is more cost-effective but may result in lower yields and the need for more efficient recovery techniques.^89^ The type of feedstock, catalyst employed, reaction temperature, and time are some variables that affect the optimal molar ratio. Advances in catalyst design and process optimization make them a potential option for sustainable and scalable biodiesel production. Homogeneous catalysts like NaOH and KOH are commonly used due to their high reactivity.^90^ On the other hand, heterogeneous catalysts such as CaO, MgO and zeolites enhanced thermal and chemical stability, reusability, and ease of separation from the reaction mixture.^91^ Heterogeneous catalysts reduce the need for extensive purification steps, lowering overall production costs and environmental impact. Metal oxides provide strong basic sites that efficiently convert triglycerides to biodiesel.^91^

### Thermochemical liquefaction

Thermochemical Liquefaction is the process whereby wet microalgal biomass is converted into bio-crude oil under moderate temperature (200–350 °C) and high pressure (5–20 MPa), most often in water or a solvent.^92^ Water acts as both a reactant and a solvent. It helps in hydrolysis and prevents excessive carbonization. Organic solvents can enhance the extraction of lipids and other hydrocarbons. The hydrogenation of bio-crude is facilitated by increased pressure, which makes gases like H_2_ more soluble in the liquid phase. Higher temperatures favor the production of bio-crude oil and gas by hydrogenation.Microalgal biomass + H_2_O → Bio-Crude + CO_2_ + Char + H_2_

Products include bio-crude oil (the liquid hydrocarbon fraction), CO_2_, solid char, and minor gases such as H_2_ and CH_4_.

Char formation during thermochemical liquefaction of microalgal biomass is a significant challenge. The accumulation of char reduces the yield of valuable bio-crude oil and poses a risk to catalyst deactivation in catalytic liquefaction processes.^93^ Char blocks the active sites of catalysts, hindering their effectiveness and reducing the rate of bio-crude production. Char formation during thermochemical liquefaction can be mitigated by optimizing the reaction temperature and time. Operating under higher pressures keeps water liquid, minimizing char production. Pre-treating biomass to remove inorganic impurities further reduces char formation.

### Gasification

Gasification converts microalgal biomass into syngas, a carbon monoxide and hydrogen gas mixture, with oxygen or air, steam, and/or carbon dioxide using high temperatures (700–1000 °C). This process is mainly used for power generation and chemical synthesis. The main products are syngas (CO and H_2_) with minor amounts of CO_2_.Microalgal biomass + O_2_ + H_2_O → CO + H_2_ + CO_2_

Operating factors significantly impact the quality of syngas generated during gasification. Higher temperatures frequently enhance the synthesis of H_2_ and CO by encouraging endothermic reactions like steam reforming, producing high-quality syngas.^94^ Although increasing the steam-to-biomass ratio encourages the creation of hydrogen, water-gas shift processes may cause the CO concentration to drop. A significant factor is the choice of gasifying agents like air, oxygen, or steam. Air gasification yields syngas with lower heating values due to nitrogen dilution. Oxygen or steam gasification produces higher quality.

### Hydrolysis and fermentation

Microalgae carbohydrate elements such as starch or cellulose are fermented with ethanol during production.^95^ The fundamental process involves hydrolysis. In hydrolysis, polysaccharides are broken into fermentable sugars, which are then fermented. Using acids or enzymes, the starch or cellulose was converted into sugars (glucose).Microalgal biomass + H_2_O → C_2_H_5_OH (Bioethanol) + 2CO_2_

During fermentation, contaminants can reduce the quantity and quality of bioethanol and create hazardous by-products. Contamination by bacteria can lead to the production of lactic acid and acetic acid, which lower bioethanol quality. Temperature and pH significantly influence the bioethanol production. Under fermentation conditions, a slightly acidic condition of 4.5–5.5 is preferred.^57^ Extreme low pH can inhibit algal metabolism and impact the biomass. During fermentation, pH has an impact on enzyme activity and microbial growth. Fermentation can be slowed if the pH is too high or too low. Temperature controls the speed of microbial reactions. High temperatures can enhance metabolic activity for bioethanol production. However, excessive heat denatures proteins and enzymes and inhibits the fermentation process.

Many factors are involved in microalgae cultivation. It is affected by pH, light, temperature, and growth media.^96,97^ Dammak *et al.* said that the ideal pH range for algal growth is 8.2 to 8.7 which can be achieved by adding CO_2._^98^ Since every type of algae has different light requirements, the light intensity and duration of light is crucial variables. To avoid contamination and promote algal growth, it is important to maintain high-quality culture media. Nutrients play a crucial role in determining microalgae growth and lipid production. The balance between all the nutrients is essential as an imbalance can inhibit lipid production and less biomass. Nitrogen and phosphorus are significant macronutrients for algal growth. Under abundant nitrogen conditions, the biomass production of algae is higher. However, high nitrogen levels may inhibit lipid synthesis as it leads to a lower lipid-to-biomass ratio.^99^ In conditions where phosphorus and iron are limited, it can stimulate high lipid production; however, it reduces chlorophyll content and overall growth.^99^ Algae are commonly cultivated using different methods such as open pond systems, photobioreactors and hybrid systems. Temperature has big impact on algal growth, finding the right regions to cultivate. Algae can be grown successfully by controlling the above mentioned parameters. In order to maximize the production of biofuel, genetic engineering and the selection of macroalgal species are essential. Species that have a high lipid and carbohydrate content are preferred for biofuel production. Enhancing microalgae-based biofuel production requires genetic traits including lipid yield, stress tolerance, and rapid growth. The generation of biofuel may be greatly increased by genetically modifying these genes in microalgae species. Optimizing rubisco (ribulose-1,5-bisphosphate carboxylase/oxygenase) promotes high growth and carbon fixation. Acetyl-CoA Carboxylase (ACCase) increases fatty acid production, which in turn enhances stress tolerance.^100^ Diacylglycerol acyltransferase (DGAT) is essential for promoting lipid buildup.^101^

### Macroalge

**Fig. 3:**
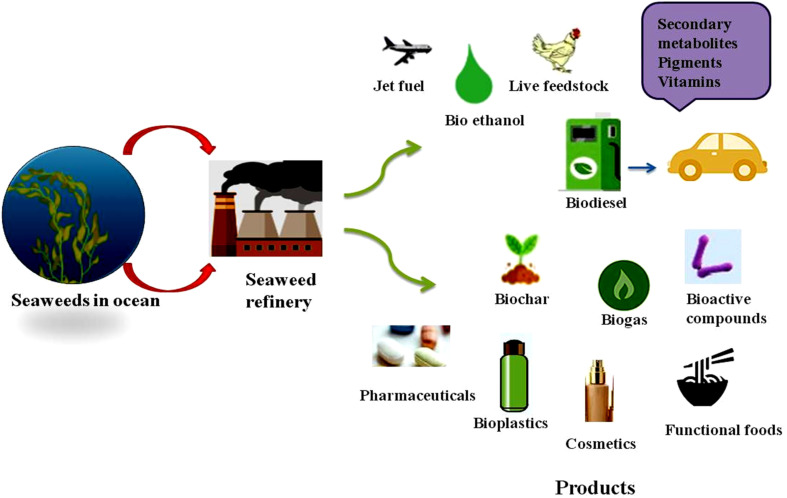
Commercial application of seaweeds and its various products.

Macroalge are broad group multi cellular autotrophs. They are found in various environments, including seagrass meadows, freshwater communities, and estuarine and marine waters.^59^ They are over 350 species including all major taxonomic divisions. They are popularly known as seaweeds.^59,60^ There cultivation condition varies from species to species as they thrive in coastal saline environments. They required optimal temperature 10–25 °C and abundant water, nutrient supply. Macroalgae are widely employed in industries such as food, fertilizer, and cosmetics, and they generate enormous economic value, particularly in Asia^102^ (Fig. 3). Seaweeds have been used for food products in Asian countries since centuries. Seaweed has the potential to provide a larger share of nutrients to the world without generating GHG emissions. Macroalgae contain polysachharides making it suitable for food as well as pharmaceutical industries. Macroalgal biomass can be used as an additive to livestock and other animal feeds, reducing enteric methane emissions, and as a diet supplement for poultry. These also have applicability in cosmetic industries due to the presence of various pigments. Macroalgae are high in carbohydrates (up to 60%), moderate to high in proteins (10–47%), and low in lipids (1–3%), with varied amounts of mineral ash (7–38%).^59,60^ The composition of carbohydrates is varying across species of seaweeds. 5–20% cellulose present for the structural support in most algae. 30–60% of carbohydrates in brown algae is alginates. The green algae composed of mannose and xylose.^103^ The brown algae have sulfated polysaccharides with fucose. They are rich in essential amino acids. But in general, lysine and methionine are present in lower quantities. Seaweed proteins are considered to be incomplete. The composition of amino acid in seaweed varies by species to species. Red algae have higher amounts of glutamic acid, aspartic acid, and glycine.^104^ The production of biofuel typically required carbohydrates and lipids. Thus, protein content and its amino acid composition have minimal impact in production of biofuels. These proteins could influence the growth of algal biomass. Hence it can indirectly affecting the overall efficiency of biofuel production. The fatty acid composition in seaweed varies by species. The major fatty acids found in seaweeds are unsaturated. The unsaturated fatty acid content is beneficial for biofuel production.^105^ High unsaturated fatty acids contribute to better fuel quality, such as lower viscosity and improved oxidation stability. Saturated fatty acids like palmitic acid and stearic acid are found in lower amount. Oleic acid is common in many species. Linoleic acid is commonly present in *Ulva*. *Laminaria* contain eicosapentaenoic acid and docosahexaenoic acid. The chemical composition of macroalgae makes it ideal for manufacturing biofuels *via* processes such as thermal treatment and fermentation.^106^

## Seaweeds: promising feedstock for biofuel production

Seaweeds are classified into three classes based on their thallus color: brown (Phaeophyceae), red (Rhodophyceae), and green (Chlorophyceae).^107^ Carbohydrates are a major component of the seaweed, comprising 25–50% of green algae and 30–60% of both red algae and brown algae. Green algae contain 10–20%, red algae contain 10–25% and brown algae contain 3–15% of protein. The lipid content is 1–4% for green algae, 0.6–4% for red algae, and 0.4–2.4% for brown algae. Biogas has been produced using anaerobic digestion from a wide range of seaweeds, including *Laminaria*, *Macrocystis*, *Gracilaria*, *Sargasum*, and *Ulva*. Biogas typically contains 50–70% methane, 30–45% carbon dioxide, less than 3.5% hydrogen sulfide, and less than 2% hydrogen.^108^ Each species of seaweed has a distinct carbohydrate profile. Brown seaweed contains alginate, fucoidan, and cellulose. Brown seaweeds, particularly the *Sargassum* species, are widely used for ethanol production in West Africa.^109^ Brown seaweed can yield up to 13.1 kg of biomass per square meter annually.^110^ Red seaweeds have polysaccharides like cellulose and mannan in their cell walls. Green seaweeds are mostly found in shallow waters. They have chlorophyll pigments and require abundant sunlight.

**Fig. 4:**
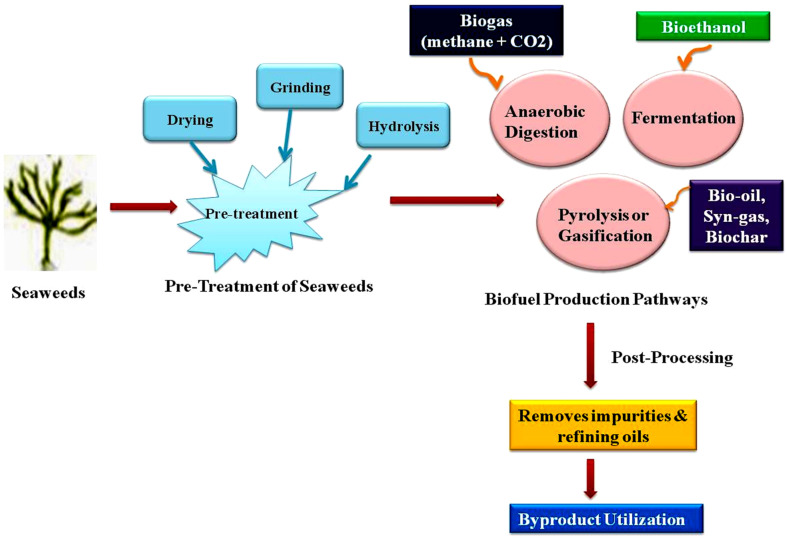
Biofuel production pathways by seaweeds in biorefinery.

Seaweeds are highly promising feedstocks for bioethanol and biogas production (Fig. 4). Due to their rapid growth, high carbohydrate content, and efficient photosynthetic activity it is also considered as an ideal source. Macroalgae contain minimal lignin, which makes them easy to process for bioethanol.^5^ These renewable resources are sulfur-free, highly biodegradable, and widely available in different species. Major cultivation areas include East Asia, where China leads production with around 14 million tones.^111^ Unlike microalgae, which require bioreactors, macroalgae can grow in open marine environments, lowering nutrient supply costs and energy use. The commercial seaweed farming involves either onshore cultivation or direct harvest. Macroalgae cultivation contributes to carbon sequestration, as they absorb large amounts of CO_2_ and reduce environmental pollution. Seaweed farming has significant potential for carbon sequestration through the absorption and storage of CO_2_ during photosynthesis. Seaweeds absorb CO_2_ from the surrounding saltwater. They create organic carbon that can be stored in the seaweed biomass. *Laminaria* and *Macrocystis* tend to have higher carbon sequestration capacity due to their thick cell walls.^112^ On the other hand, because of their smaller size and weaker cell walls, red and green algae tend to store less carbon. Perennial brown algae could deplete around 10 tons of CO_2_ per hectare of marine surface each year. Seaweed farming can also remove inorganic nutrients in the seas, reduce nitrogen and phosphorous pollution. Macroalgae cell wall is rich in polysaccharides which make them well-suited for bioethanol production.^5^ Macroalgae can cleanse water by removing heavy metals, and their cultivation helps increase ocean oxygen, benefiting the environment. Another growing interest is the search for alternative proteins derived from terrestrial livestock under conventional production technologies. Because of their nutritional and functional qualities, alternative proteins made from seaweed are becoming more and more popular. These can be wholesome choice for food and feed applications. They are rich in fiber, vitamins, and minerals. Hence, they are used in a variety of culinary items, including snacks, dairy alternatives, and meat analogs.^113^ Unlike animal-derived proteins, seaweed proteins are free from cholesterol. When compared to plant-based proteins, they have lower protein content but concentrations of minerals and bioactive substances.

### Emerging routes of biofuel production from seaweed biorefinery

Similar to petroleum refineries, biorefinery aim to convert biomass into biofuels, chemicals, feed materials, and other value-added products (Fig. 4). These processes are designed to minimize waste and environmental impact within a fully integrated and highly efficient processing system. In biorefinery, biomass is utilized at each step of the integrated process to extract value-added compounds. The goal is to achieve nearly zero waste by efficiently using all bioresources, including the biological remains generated during the cascade transformation of biomass (Fig. 4).

Biorefineries convert biomass in a cascade process into a variety of useful products. The average outputs include 100–400 L of bioethanol per dry ton of lignocellulosic biomass, 100–150 L of biodiesel per hectare per year from algae, and 100–150 m^3^ of biogas per ton of organic waste. Petroleum refineries are converting up to 70–80% of crude oil into usable products, such as 40–50% gasoline and 20–30% diesel. Biorefineries provide substantial sustainability advantages and the potential to lower greenhouse gas emissions despite their lower energy density and product yield efficiency.^114^

Steps of preparation of seaweeds before pre-treatment

• Washing: removal of all unnecessary objects like sand, salts, stones, debris, or trash.

• Drying: dewatering the seaweed is the best possible way for future conversion and application. It increases stability while decreasing volume during storage and processing.

• Milling: reduce the size of the macroalgae in the order of millimeters to increase the surface area.

Different types of pretreatments employed for the production of biofuels are as follows:

• Milling and extrusion: reduce the size of particles by breaking. It facilitates improved accessibility in posterior enzymatic processes, minimizing the production of inhibitory compounds and by-products.

• Ball milling: enhance the conservation efficiency of seaweed into biogas.

• Vibro-ball and centrifugal milling: improve enzymatic saccharification of polysaccharides from *Ulva lactuca* and *G. sesquipedate*.

• Microwave: used as another heating to thermal treatment of biomass due to the speed and selectivity of the method.

• Combination with acid, alkali solvents, ionic liquids (imidazolium) to enhance effectiveness.

Ionic liquids are effective pretreatment agents for seaweed biomass by disrupting its cell wall structure. By dissolving complex carbohydrates like cellulose and alginate, Ionic liquids ILs weaken the inter- and intra-molecular bonds.^115^ It depolymerizes long polysaccharide chains into smaller fragments. They also solvate cellulose, hemicelluloses and remove lignin and proteins that may inhibit enzymatic activity. Ionic liquids are potential boosting agents for biofuel production from seaweed because these processes improve the surface area and accessibility of polysaccharides for enzymatic saccharification.

Coastal integrated marine biorefinery (CIMBs) use resources like seawater and marine microorganisms for biofuel production. These systems are highly resource-efficient because they do not need freshwater.^116^ While processing biomass, CO_2_ is trapped and aids in efforts to sequester carbon. Advanced types called coastal integrated marine biorefinery combine seaweed and microalgae processes. CIMBs enhance production efficiency by optimizing outputs and reducing waste.^117^ These systems store energy, produce renewable electricity, and promote economic growth in rural coastal regions. The CIMB ensures resource efficiency and reduces environmental impact by combining many phases of biomass conversion. It improves sustainability and minimizes waste by utilizing renewable energy sources and maximizing marine biomass production. While scaling up CIMBs involves high initial capital costs for infrastructure and technology, the long-term benefits include reduced reliance on fossil fuels and mitigating coastal eutrophication.^114^ Continuous cost reductions and technology advancements are necessary for commercial success.

Seasonal and geographical variability in macroalgal biomass production also affect biofuel production. The seasonal fluctuations of variables include temperature, light availability, salinity, and nutrient levels.^118^ Macroalgae have lower biomass accumulation in adverse environmental conditions, affecting biofuel production. Because of this fluctuation, it may be challenging to supply macroalgal biomass for biofuel manufacture. In order to improve yield and lessen reliance on outside environmental conditions, methods like selective breeding and genetic change practices are crucial. The genetic engineering of seaweeds for improved biofuel production focuses on characteristics such as biomass yield, growth rate, and efficiency. Higher biomass yields can also be achieved by altering nitrogen and carbon consumption pathways.^99^ Modifying targeting genes involved in fatty acid synthesis could enhance lipid accumulation for biodiesel production. Improving stress tolerance by adjusting heat shock proteins or osmotic regulators may maintain consistent biomass production. However, introducing genetically modified seaweeds into natural ecosystems poses ecological risks. It might disturb the original biodiversity.

Integrating microalgal and macroalgal biomass production with existing infrastructure, such as wastewater treatment plants and agricultural facilities, offers significant environmental and economic benefits.^119^ Algae can absorb excess nutrients from wastewater, especially nitrogen and phosphorus. Therefore, water quality is improved while valuable biomass for biofuels is produced. Additionally, this integration helps lessen the adverse environmental effects of wastewater outflow and agricultural runoff by using waste products and lowering the demand for external fertilizer inputs. The integration also poses several difficulties, such as the requirement for considerable infrastructure modifications to support algae production systems. A significant investment may be needed to scale up to meet high biofuel demands in these existing infrastructures.

### Economic challenges in algal biofuel production

Despite these benefits, there are obstacles to the commercialization of algal biofuels. Algae-derived biofuels encounter considerable difficulties in competition with petroleum. In order to be a feasible substitute for petroleum, algae biofuels must address challenges regarding algae cultivation. There are still obstacles to expanding production and enhancing the economic feasibility of algal biofuels. Producing a barrel of algae-based fuel costs between $ 300 and $ 2,600, compared to just $ 40 to $ 80 for petroleum.^10^ Strain isolation and other physiological factors must be addressed to make algae-based biofuels economically competitive.^12^ The scalability and economic viability of algae-based biofuels hinge on overcoming challenges like optimizing cultivation, reducing extraction costs and refining fuel processing techniques. The adoption of algae-based biofuels faces several key barriers. Scaling up algae biofuel production from laboratory to commercial scale involves several technological challenges. Open pond and photobioreactor systems required for large-scale algal cultivation. But to, setting it up on large scale involves high initial capital investments and substantial ongoing operational costs.^120^ Algal growth in such systems is highly sensitive to environmental fluctuations. Harvesting and dewatering algae are typically energy-intensive process. Although some regions report lower costs, the general trend indicates that algae oil is not yet competitive in the current liquid fuel market. Bio-flocculation and membrane filtration are two low-energy harvesting strategies that have been developed. Furthermore, integrating technologies like hydrothermal liquefaction and pyrolysis into algal biorefineries improves overall efficiency.^121^ Genetic engineering and metabolic optimization are being used to improve the accumulation of lipids and carbohydrates in algal cells. The lack of clear regulatory policies hinders market entry and investment. Government and industry partnerships must also work to implement supportive policies. By encouraging innovation in low-energy processing technologies, collaborative research and development can help overcome these obstacles.

## Conclusion

Biofuel production has grown significantly. First-generation biofuels face challenges such as competition with food production and higher costs. In contrast, second-generation biofuels avoid food supply issues but increase land-use efficiency. Third and fourth-generation biofuels also show promise but are still under research. The production of biofuels from algae is still in its developmental stages, but significant progress has been made in culturing techniques, oil extraction methods, and hybrid systems, enhancing efficiency and cost-effectiveness. Algae have capacity to grow on non-arable soil and absorb waste streams increases its potential as a sustainable biofuel source. Furthermore, breakthroughs in genetic engineering are improving algal biofuel production, making it a viable solution for future energy needs. Algae's ability to grow quickly, consume CO_2_ efficiently, and produce high lipid content makes them an appealing feedstock for biofuels. These methods embody sustainable processes whereby safer alternatives could emerge for traditional methods. Presently, however, difficulties arise in escalating output rates and increasing the commercial feasibility of algal-based biofuels. Continuous research and development of algal biofuels and genetically modified algae technologies will be crucial. A hybrid system that combined both open ponds and photobioreactors appears to hold the best promise to increase productivity while remaining cost-effective. The huge potential of algae as a biofuel feedstock must, however, be proven through additional technical efforts.

## Author contributions

D P Krishna Samal: conceptualization, writing – original draft, data analysis, formatting. Lala Behari Sukla: supervision, editing.

## Conflicts of interest

The authors declare that they have no known competing financial interests or personal relationships that could have appeared to influence the work reported in this paper.

## Data Availability

The data supporting the findings of this study are available from the corresponding author upon reasonable request. Please feel free to contact me on lalabeharisukla@soa.ac.in, if any required for further details or access to the data.
